# Synthesis and oxidation of some azole-containing thioethers

**DOI:** 10.3762/bjoc.7.179

**Published:** 2011-11-16

**Authors:** Andrei S Potapov, Nina P Chernova, Vladimir D Ogorodnikov, Tatiana V Petrenko, Andrei I Khlebnikov

**Affiliations:** 1 Department of Chemistry, Altai State Technical University, 46 Lenin Ave., 656038 Barnaul, Russia; 2 Institute of Petroleum Chemistry, Siberian Branch of the Russian Academy of Sciences, 3 Akademicheskii Ave., 634055 Tomsk, Russia

**Keywords:** azole, oxidation, sulfone, sulfoxide, thioether

## Abstract

Pyrazole and benzotriazole-containing thioethers, namely 1,5-bis(3,5-dimethylpyrazol-1-yl)-3-thiapentane, 1,8-bis(3,5-dimethylpyrazol-1-yl)-3,6-dithiaoctane and 1,3-bis(1,2,3-benzotriazol-1-yl)-2-thiapropane were prepared and fully characterized. Oxidation of the pyrazole-containing thioether by hydrogen peroxide proceeds selectively to provide a sulfoxide or sulfone, depending on the amount of oxidant used. Oxidation of the benzotriazole derivative by hydrogen peroxide is not selective, and sulfoxide and sulfone form concurrently. Selenium dioxide-catalyzed oxidation of benzotriazole thioether by H_2_O_2_, however, proceeds selectively and yields sulfoxide only.

## Introduction

Compounds comprising two pyrazole moieties linked by an aliphatic spacer act as bidentate chelating ligands, forming complexes with most transition metals and some main-group elements [1]. The coordinating ability of these ligands can be diversified by the introduction of additional donor atoms into the spacer between the heterocycles. Ligands with spacers bearing nitrogen, oxygen, and sulfur atoms have been reported, and some of them were found to be effective inhibitors of steel corrosion [2], while their chromium(III) and palladium(II) complexes demonstrated catalytic activity in ethylene oligomerization [3] and Heck cross-coupling reactions [4]. Recently we and others have reported high superoxide dismutase-like activity of copper(II) complexes with bis(pyrazole) ligands [5–6]. Copper(II) complexes with azole-derived thioether ligands were proposed as models for type I copper proteins [7]. The sulfur atom in a thioether spacer gives an additional possibility for modification of the ligand structure through its conversion to sulfoxide or sulfone derivatives. In this report we present the synthesis of pyrazole and benzotriazole thioether ligands and their oxidation to the corresponding sulfoxides and sulfones.

## Results and Discussion

**Scheme 1:**
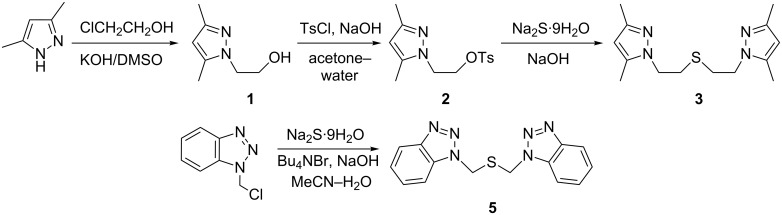
Synthesis of azole-containing thioethers.

1-(2-Hydroxyethyl)-3,5-dimethylpyrazole (**1**), the starting compound for the synthesis of pyrazole-containing thioether, 1,5-bis(3,5-dimethylpyrazol-1-yl)-3-thiapentane (**3**), was prepared by the reaction of 3,5-dimethylpyrazole with 2-chloroethanol in a superbasic potassium hydroxide–dimethylsulfoxide system (Scheme 1). This procedure does not employ toxic solvents, expensive reagents or catalysts in contrast to methods reported by others [8–10].

The preparation of pyrazole-containing thioether, 1,5-bis(3,5-dimethylpyrazol-1-yl)-3-thiapentane was reported previously by Sorrel et al. [11] and Haanstra et al. [12]. The method used by Sorrel involves the reaction of 1-(2-chloroethyl)-3,5-dimethylpyrazole hydrochloride with sodium sulfide in 50% aqueous ethanol. The product was obtained in the form of an oil, even after purification by flash chromatography [11]. Haanstra et al. used the tosyloxy derivative instead of the chloro derivative in the reaction with sodium sulfide. The thioether was isolated as a crystalline powder, but it was used without purification as a ligand for complex preparation, so no analytical data was reported, except for the ^1^H NMR spectrum [12]. Ros et al. proposed an alternative synthetic route starting from 1-(2-mercaptoethyl)-3,5-dimethylpyrazole and fully characterized the thioether for the first time [13]. We found that the use of freshly recrystallized Na_2_S·9H_2_O in the reaction with 1-(2-hydroxyethyl)-3,5-dimethylpyrazole tosylate (**2**) yields a solid product in good yield.

**Scheme 2:**
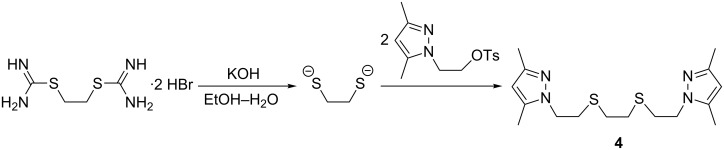
Synthesis of dithioether.

We also prepared a pyrazole-containing thioether **4** with two sulfur atoms by the reaction of tosylate **2** with 1,2-ethanedithiolate formed in situ from diisothiuroium salt of 1,2-dibromoethane (Scheme 2). The proposed method of synthesis has an advantage over the previously reported one [8], since it does not involve the use of odoriferous and unstable 1,2-ethanedithiol.

The benzotriazole-containing thioether **5** was prepared following a similar strategy to that for product **3** by using 1-chloromethylbenzotriazole as electrophile (Scheme 1). The mixture of aqueous Na_2_S and NaOH solution with acetonitrile is biphasic, and we found that the addition of a phase-transfer catalyst expedites the nucleophilic substitution and reduces the formation of side-products.

The formation of compound **5** was observed earlier by Katritzky et al. as a side product (11% yield) during the preparation of 1-mercaptomethylbenzotriazole [14]. The product yield achieved by the procedure proposed here was 89%, and no formation of the mercapto-derivative was detected.

The properties of azole-containing thioether can be varied by functionalization of the azole ring or oxidation of the sulfur atom, and therefore we have studied the reactivity of thioethers **3**–**5** in electrophilic substitution and oxidation reactions.

Oxidation of thioethers to sulfoxides and sulfones can be achieved by using different oxidants and catalysts [15], hydrogen peroxide being the most versatile and green among them [16]. Despite the simplicity of their preparation and potentially useful properties, azole-containing sulfoxides and sulfones are not described in the literature.

**Scheme 3:**
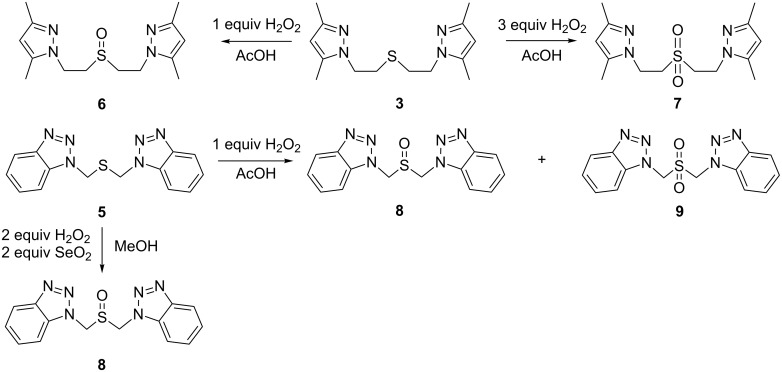
Oxidation of thioethers to sulfoxides and sulfones.

The action of one mole of hydrogen peroxide on thioether **3** in acetic acid at room temperature for 2.5 h selectively gives sulfoxide **6** (85% yield, Scheme 3). Raising the temperature to the boiling point of acetic acid (118 °C) and using an excess of an oxidant results in the formation of sulfone **7** (77% yield, Scheme 3).

Benzotriazole-containing thioether **5** is considerably less reactive in the oxidation reaction promoted by hydrogen peroxide in acetic acid. Thus, upon addition of one equivalent of hydrogen peroxide, only the starting compound **5** was isolated, even after 96 h at room temperature. Increasing the quantity of H_2_O_2_ to 1.5 equiv resulted in the formation of sulfoxide **8**, but full conversion of the starting material could not be achieved (Table 1, entry 1). Raising the temperature to 60 °C led to a considerable increase in the reaction rate and a comparable conversion was achieved in 6 h (Table 1, entry 2). Despite the use of a stoichiometric amount of H_2_O_2_, oxidation of thioether **5** or sulfoxide **8** proceeds concurrently, and a small quantity of sulfone **9** was detected in the reaction mixture. Therefore, further increase of the reaction duration in order to achieve greater conversion seemed impracticable, since that would lead to formation of a sulfone and sulfoxide mixture. Although the conversion was incomplete, isolation of sulfoxide **8** from the reaction mixture was quite simple, since sulfoxide **8** precipitates from the solution over the course of the reaction. Thus, the precipitate obtained in one of the oxidation runs (Table 1, entry 2) contained 89% sulfoxide **8** by volume, while the remaining solution contained only 30% **8** by volume. Crystallization of the precipitate from DMF gave pure sulfoxide **8**.

**Table 1:** Conditions and product mixture composition for oxidation reaction of thioether **5**.

Entry	Oxidant	Temperature (°C)	Time (h)	Product composition (by NMR)		
				thioether **5**	sulfoxide **8**	sulfone **9**

1	H_2_O_2_ (1.5 equiv)	20	84	37	63	traces
2	H_2_O_2_ (1 equiv)	60	6	41	58	1
3	H_2_O_2_ (4 equiv)	95	48	–	44	56
4	H_2_O_2_ (4 equiv)	118	24	–	–	100
5	H_2_O_2_ (1 equiv), SeO_2_ (1 equiv)	20	3	87	23	–
6	H_2_O_2_ (1 equiv), SeO_2_ (1 equiv)	60	3	43	56	1
7	H_2_O_2_ (1 equiv), SeO_2_ (1 equiv)	60	24	42	55	3
8	H_2_O_2_ (2 equiv), SeO_2_ (2 equiv)	60	6	5	86	9

Oxidation of sulfoxide **8** to sulfone **9** is even more complicated than the oxidation of thioether **5** to **8**. Even at 95 °C and with a two-fold excess of H_2_O_2_, oxidation to sulfone **9** proceeds only to 56% (Table 1, entry 3), and a full oxidation could only be achieved by refluxing the reaction mixture in acetic acid for 24 h (Table 1, entry 4).

It is evident that hydrogen peroxide alone is not an effective oxidant for thioether **5**, therefore other oxidizing systems should be evaluated. Selenium(IV) oxide was reported to be an efficient catalyst in a thioether oxidation reaction promoted by H_2_O_2_ [17]. However, the performance of the H_2_O_2_/SeO_2_ system has only been studied on a few substrates, and thus we decided to evaluate it for thioether **5** oxidation. It was found that the addition of the SeO_2_ catalyst results in about a two-fold increase of the oxidation rate (Table 1, entries 5, 6, compared with entry 2). In addition, when catalyzed by SeO_2_, the oxidation becomes more selective and gives sulfoxide **8** with only a small amount of sulfone, even when an excess of reagents is used (Table 1, entry 8). Therefore, due to its selectivity, the H_2_O_2_/SeO_2_ system proved to be very useful for the preparation of sulfoxide **8** from the unreactive compound **5**.

**Scheme 4:**
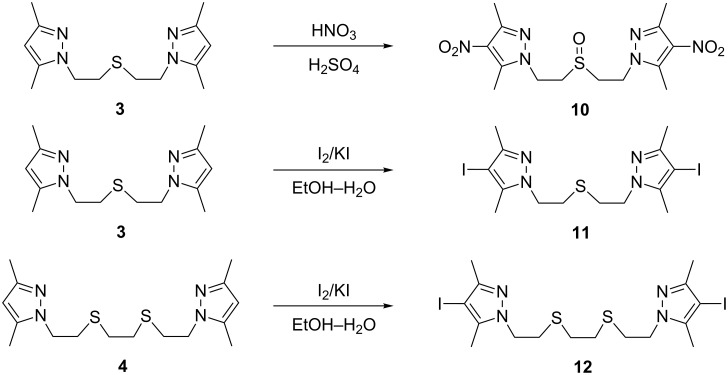
Preparation of functional derivatives.

The reactivity of pyrazole-containing thioether **3** in aromatic electrophilic substitution was evaluated by using nitration and iodination reactions as examples. Nitration of compound **3**, by 68% nitric acid in concentrated sulfuric acid, results in both substitution in the heterocyclic rings and sulfur atom oxidation to sulfoxide (Scheme 4).

Previously, we found that the oxidative iodination of different bis(pyrazole) compounds proceeds smoothly when using an I_2_/HIO_3_ system [18]. The action of this system on thioethers **3** and **4** resulted in incomplete iodine consumption and a complex product mixture, which contained, according to NMR, both mono- and disubstituted iododerivatives of the corresponding sulfoxides together with the starting material. Obviously, the presence of a reductive sulfur atom in the substrate deactivates the iodinating system, and therefore oxidative iodination is unsuitable for thioether functionalization. Using a non-oxidative aqueous I_2_/KI system [19] readily yields the corresponding diiodo-thioethers **11** and **12** (Scheme 4).

**Figure 1:**
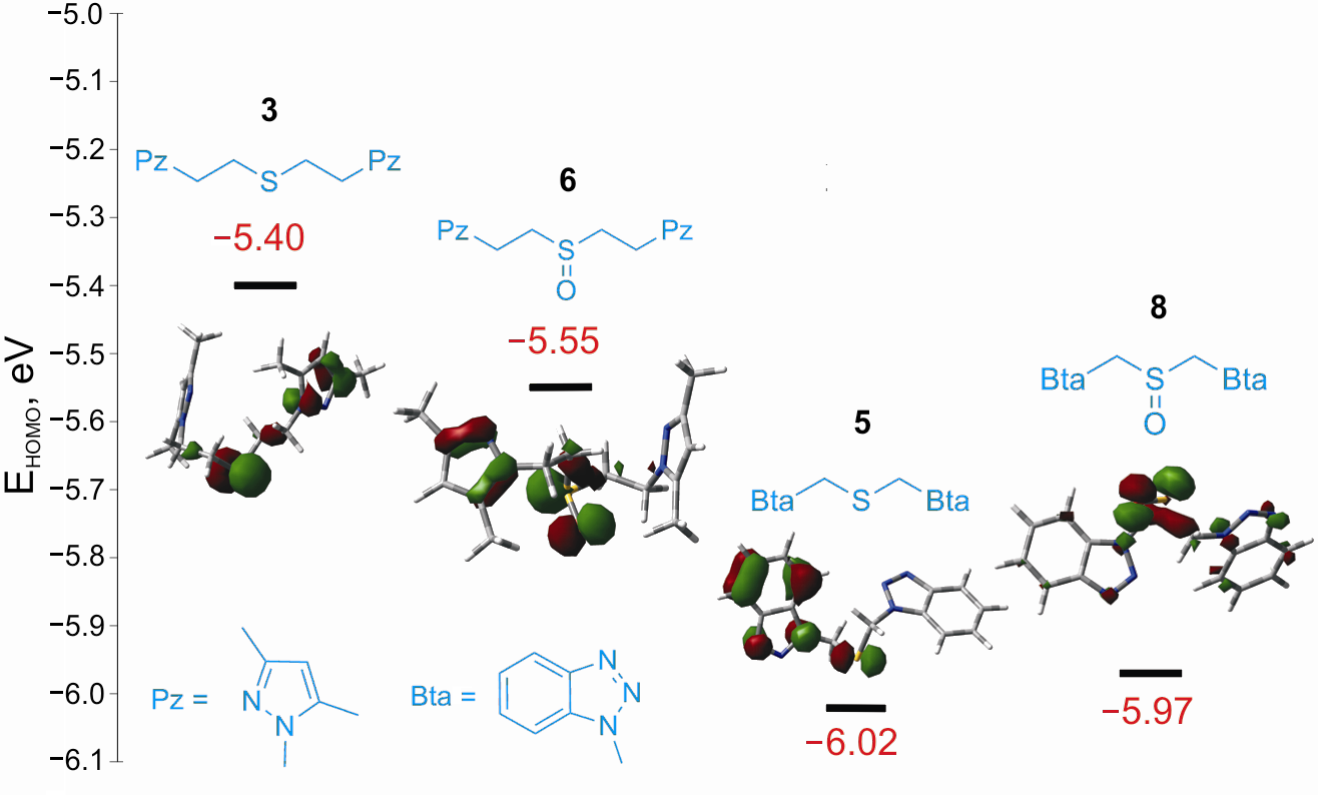
Energies and isosurfaces of the highest occupied molecular orbitals (HOMO) of azole-containing thioethers and sulfoxides according to DFT calculations (BP86 functional, TZVPP basis set).

In order to explore the reasons for the different reactivity of thioethers **3** and **5** in the oxidation reaction, we carried out DFT calculations of the electronic structure of these compounds and their corresponding sulfoxides. Ionization energies were evaluated according to Koopman’s theorem [20] as a negative of the HOMO energies. The energy diagram showing relative HOMO energies and orbital isosurfaces is presented in Figure 1.

As can be seen from Figure 1, the HOMO energy of the benzotriazole-derived thioether **5** is lower than that of the pyrazole-containing compound **3**; moreover, in the latter compound the majority of the HOMO electronic density is located on the sulfur atom, while in compound **5** it is more delocalized on the electron-withdrawing benzotriazole cycle. These peculiarities of the HOMO lead to a greater reactivity of the pyrazole-derived thioether **3** compared to the benzotriazole derivative **5**.

The lower selectivity in the oxidation of the benzotriazole-containing thioether **5** is probably explained by the comparable HOMO energies of compounds **8** and **5** (Figure 1), especially as the HOMO energy of sulfoxide **8** is even slightly higher, leading to its higher reactivity in oxidation reactions.

## Conclusion

In summary, we have prepared new pyrazole- and benzotriazole-derived sulfones and sulfoxides that are interesting in view of their coordination chemistry and their potential biological activity. The lower reactivity and selectivity of benzotriazole-containing thioether in oxidation reactions is explained by the presence of electron-withdrawing heterocycles in proximity to the sulfur atom, but nevertheless, the corresponding sulfoxide or sulfone can be selectively isolated by varying the oxidation conditions. Sulfone can be prepared by the addition of an excess of H_2_O_2_ in acetic acid at elevated temperature, while sulfoxide is selectively formed upon the addition of H_2_O_2_ and selenium dioxide in methanol.

## Experimental

### General methods

Elemental analyses were carried out on a Carlo Erba analyzer. Infrared (IR)-spectra of solid samples as KBr pellets were recorded on a Nicolet 5700 (4000–400 cm^−1^) spectrophotometer. NMR spectra were recorded on a Bruker AV300 operating at 300 MHz for ^1^H and 75 MHz for ^13^C.

### Computational chemistry details

A conformational search by means of the semi-empirical PM3 method was performed for thioethers **3**, **5** and sulfoxides **6**, **8**, taking into account rotations around all single bonds (C–C, C–S, C–N). Minimal energy conformations were used as starting points for further, more accurate geometry optimizations at the RI DFT BP86 [21] level of theory and with the TZVPP [22] basis set (TZV/J auxiliary basis set [23–24]). Frequency calculations were run in order to establish the nature of the stationary points. The lack of imaginary vibration modes for all of the optimized structures indicates that the stationary points found correspond to minima on the potential-energy surface. Calculations were performed with the ORCA 2.8.0.2 package [25].

### Experimental procedures and analytical data for new compounds

**1,1'-(Sulfinylbis(ethane-2,1-diyl))bis(3,5-dimethylpyrazole) (6)**. To thioether **3** (0.3 g, 1.08 mmol) in acetic acid (1 mL), hydrogen peroxide (0.075 mL of 50% aqueous solution, 1.08 mmol) was added. After being stirred for 2 h at room temperature, the reaction mixture was poured into water (15 mL) and extracted with chloroform (5 × 10 mL). The extract was washed with water (2 × 10 mL), dried over calcium chloride and the solvent was evaporated in vacuo. Yield 0.27 g (85%), colorless crystals, mp 130–131 °С (benzene); IR (cm^−1^): 1549, 1461, 1428, 1315 (Рz), 1021 (S=O), 789 (С–S); ^1^Н NMR (CDCl_3_) δ 2.16, 2.23 (s, 12Н, СН_3_), 3.10 (ABXY m, 4Н, PzCH_2_C*H*_2_SО), 4.33 (ABXY m, 4Н, PzC*H*_2_CH_2_SО), 5.74 (s, 2Н, Н4 (Pz)); ^13^С NMR (CDCl_3_) δ 10.7 (5-СН_3_), 13.3 (3-СН_3_), 40.9 (PzCH_2_*C*H_2_SО), 51.9 (Pz*C*H_2_CH_2_SО), 105.1 (С4 (Pz)), 139.5 (С5 (Pz)), 148.3 (С3 (Pz)); Anal. calcd for С_14_Н_22_N_4_OS: С, 57.11; Н, 7.53; N, 19.03; S, 10.89; found: С, 57.50; Н, 7.60; N, 19.15; S, 10.69.

**1,1'-(Sulfonylbis(ethane-2,1-diyl))bis(3,5-dimethylpyrazole) (7)**. To thioether **3** (0.87 g, 3.4 mmol) in acetic acid (1 mL), hydrogen peroxide (0.64 mL of 50% aqueous solution, 9.42 mmol) was added. After being heated under reflux for 5 h, the reaction mixture was poured into water (25 mL) and extracted with chloroform (8 × 10 mL). The extract was washed with water (2 × 10 mL), dried over calcium chloride and the solvent was evaporated in vacuo. Yield 0.75 g (77%), colorless crystals, mp 122–123 °С (toluene); IR (cm^−1^): 1552, 1466, 1390 (Рz), 1298, 1128 (О=S=O), 772 (С–S); ^1^Н NMR (CDCl_3_) δ 2.15, 2.23 (s, 12Н, СН_3_), 3.24 (t, *J* = 6 Hz, 4Н, PzCH_2_C*H*_2_SO_2_), 4.33 (t, *J* = 6 Hz, 4Н, PzC*H*_2_CH_2_SO_2_), 5.76 (s, 2Н, Н4 (Pz)); ^13^С NMR (CDCl_3_) δ 10.7 (5-СН_3_), 13.3 (3-СН_3_), 41.3 (PzCH_2_*C*H_2_SO_2_), 53.1 (Pz*C*H_2_CH_2_SO_2_), 105.5 (С4 (Pz)), 139.9 (С5 (Pz)), 148.7 (С3 (Pz)); Anal. calcd for С_14_Н_22_N_4_O_2_S: С, 54.17; Н, 7.14; N, 18.05; S, 10.33; found: С, 53.98; Н, 7.05; N, 18.35; S, 10.33.

**1,1'-(Sulfinylbis(methylene))bis(benzotriazole) (8)**. Thioether **5** (0.209 g, 0.706 mmol), selenium dioxide (0.157 g, 1.41 mmol) and hydrogen peroxide (0.1 mL of 50% aqueous solution, 1.41 mmol) in methanol (4 mL) were stirred at 60 °C for 6 h. Subsequently, the reaction mixture was poured into water (20 mL), and the precipitate was filtered, washed with water and dried. Yield 0.173 g (79%), colorless crystals, mp 229–230 °C (DMF); IR (cm^−1^): 1614, 1593, 1494, 1057 (S=O), 768 (C–S), 748 (Bta); ^1^Н NMR (DMSO-*d*_6_) δ 6.41 (AB q, *J*_AB_ = 13.7 Hz, 2H, CH_2_, Δν_AB_ 0.26 ppm), 7.47 (t, *J* = 7.5 Hz, 2H, H5 (Bta)), 7.63 (t, *J* = 7.5 Hz, 2H, H6 (Bta)), 7.93 (d, *J* = 8.1 Hz, 2H, H4 (Bta)), 8.12 (d, *J* = 8.1 Hz, 2H, H7 (Bta)); ^13^С NMR (DMSO-*d*_6_) δ 62.8 (CH_2_), 111.1 (C7 (Bta)), 119.3 (C4 (Bta)), 124.6 (C5 (Bta)), 128.1 (C6 (Bta)), 133.9 (C8 (Bta)), 145.1 (C9 (Bta)); Anal. calcd for C_14_H_12_N_6_OS: С, 53.83; Н, 3.87; S, 10.27; found: С, 53.75; Н, 4.00; S, 9.86.

**1,1'-(Sulfonylbis(methylene))bis(benzotriazole) (9)**. Thioether **5** (0.457 g, 1.54 mmol) and hydrogen peroxide (0.35 mL of 50% aqueous solution, 6.16 mmol) in acetic acid (5 mL) were refluxed for 24 h. Then the reaction mixture was poured into water (30 mL), and the precipitate was filtered, washed with water and dried. Yield 0.303 g (60%), colorless crystals, mp 204–205 °C (with decomposition); IR (cm^−1^): 1613, 1592, 1494 (Bta), 1344, 1146 (O=S=O), 752 (C–S), 747 (Bta); ^1^Н NMR (DMSO-*d*_6_) δ 6.75 (s, 2H, CH_2_), 7.49 (t, *J* = 7.5 Hz, 2H, H5 (Bta)), 7.66 (t, *J* = 7.5 Hz, 2H, H6 (Bta)), 7.98 (d, *J* = 8.1 Hz, 2H, H4 (Bta)), 8.15 (d, *J* = 8.1 Hz, 2H, H7 (Bta)); ^13^С NMR (DMSO-*d*_6_) δ 63.2 (CH_2_), 111.2 (C7 (Bta)), 119.4 (C4 (Bta)), 124.8 (C5 (Bta)), 128.5 (C6 (Bta)), 133.6 (C8 (Bta)), 145.0 (C9 (Bta)); Anal. calcd for C_14_H_12_N_6_O_2_S: С, 51.21; Н, 3.68; N, 25.59; found: С, 51.63; Н, 4.02; N, 25.98.

**1,1'-(Sulfinylbis(ethane-2,1-diyl))bis(3,5-dimethyl-4-nitropyrazole) (10)**. To thioether **3 (**0.647 g, 2.33 mmol), НNO_3_ (1.61 mL of 68% aqueous solution, 23.3 mmol) and Н_2_SO_4_ (8 mL of 96% aqueous solution) were added. After the mixture was allowed to stand for 24 h at room temperature, water (50 mL) was added to the reaction mixture, and the solution was extracted by chloroform (5 × 10 mL). The extract was washed with water to neutral reaction, dried over calcium chloride and the solvent was evaporated in vacuo. Yield 0.71 g (84%), colorless crystals, mp 147–148 °С (iPrOH); IR (cm^−1^): 1561 (N–O), 1490, 1419 (Рz), 1356 (N–O), 1044 (S=O); ^1^Н NMR (CDCl_3_) δ 2.47, 2.66 (s, 12Н, CН_3_), 3.25 (ABXY m, 4Н, PzCH_2_C*H*_2_SО), 4.48 (ABXY m, 4Н, PzC*H*_2_CH_2_SО); ^13^С NMR (CDCl_3_) δ 11.2 (5-СН_3_), 13.9 (3-СН_3_), 41.9 (PzCH_2_*C*H_2_SО), 51.1 (Pz*C*H_2_CH_2_SО), 130.9 (С4 (Pz)), 140.8 (С5 (Pz)), 146.7 (С3 (Pz)); Anal. calcd for С_14_Н_20_N_6_O_5_S: С, 43.73; Н, 5.24; N, 21.86; S 8.34; found: С, 44.06; Н, 5.23; N, 21.46; S, 8.26.

**1,5-Bis(4-iodo-3,5-dimethylpyrazol-1-yl)-3-thiapentane (11)**. Thioether **3** (0.278 g, 1 mmol), sodium acetate (0.328 g, 4 mmol), potassium iodide (1.99 g, 12 mmol) and iodine (1.02 g, 4 mmol) were dissolved in the mixture of water (5 mL) and ethanol (2.5 mL) and were allowed to stand overnight at room temperature. The formed precipitate was filtered and thoroughly washed with water and Na_2_SO_3_ solution to remove traces of unreacted iodine and was then dried. Yield 0.384 g (72%), colorless crystals, mp 96–97 °C (iPrOH). Extraction of the filtrate with chloroform gives additional 0.112 g of product **11**, overall yield 94%; IR (cm^−1^): 1530, 1438, 1379, 1059 (Pz), 785 (C–S); ^1^Н NMR (CDCl_3_) δ 2.19, 2.26 (s, 12Н, СН_3_), 2.79 (t, *J* = 6.3 Hz, 4Н, PzCH_2_C*H*_2_S), 4.13 (t, *J* = 6.3 Hz, 4Н, PzC*H*_2_CH_2_S); ^13^С NMR (CDCl_3_) δ 11.9 (5-СН_3_), 13.9 (3-СН_3_), 32.0 (PzCH_2_*C*H_2_S), 49.5 (Pz*C*H_2_CH_2_S), 62.6 (С4 (Pz)), 140.6 (С5 (Pz)), 149.6 (С3 (Pz)); Anal. calcd for C_14_H_20_I_2_N_4_S: С, 31.71; Н, 3.80; N, 10.57; S, 6.05; found: С, 31.96; Н, 3.90; N, 10.23; S, 6.30.

**1,8-Bis(4-iodo-3,5-dimethylpyrazol-1-yl)-3,6-dithiaoctane (12)** was prepared similarly to compound **11**. Yield 64%, colorless crystals, mp 133–135 °C (EtOH); IR (cm^−1^): 1530, 1479, 1382, 1060 (Pz), 796 (C–S); ^1^Н NMR (CDCl_3_) δ 2.19, 2.30 (s, 12Н, СН_3_), 2.51 (s, 4H, SCH_2_CH_2_S), 2.90 (t, *J* = 6.9 Hz, 4Н, PzCH_2_C*H*_2_S), 4.33 (t, *J* = 6.9 Hz, 4Н, PzC*H*_2_CH_2_S); ^13^С NMR (CDCl_3_) δ 12.0 (5-СН_3_), 13.8 (3-СН_3_), 32.0, 32.1 (SCH_2_CH_2_S and PzCH_2_*C*H_2_S), 49.8 (Pz*C*H_2_CH_2_S), 62.6 (С4 (Pz)), 140.6 (С5 (Pz)), 149.5 (С3 (Pz)); Anal. calcd for C_16_H_24_I_2_N_4_S_2_: С, 32.55; Н, 4.10; N, 9.49; found: С, 32.36; Н, 4.30; N, 9.27.

## Supporting Information

File 1 Experimental procedures, spectroscopic and analytical data for compounds **1**, **3**–**5**.
