# Supplementary material for: Synthesis and oxidation of some azole-containing thioethers
Source: Beilstein J Org Chem. 2011 Nov 16;7:1526–32. doi: 10.3762/bjoc.7.179 (PMC3252854; doi:10.3762/bjoc.7.179)
Supplement: File 1 — Experimental procedures, spectroscopic and analytical data for compounds 1, 3–5. [file Beilstein_J_Org_Chem-07-1526-s001.pdf]

Supporting Information

for

Synthesis and oxidation of some azole-containing thioethers

Andrei S. Potapov[*1], Nina P. Chernova[1], Vladimir D. Ogorodnikov[2], Tatiana V. Petrenko[2] and Andrei I. Khlebnikov[*1]

Address: [1]Department of Chemistry, Altai State Technical University, 46 Lenin Ave., 656038 Barnaul, Russia and [2]Institute of Petroleum Chemistry, Siberian Branch of Russian Academy of Sciences, 3 Akademicheskii Ave., 634055 Tomsk, Russia

Email: Andrei I. Khlebnikov - aikhl@chem.org.ru; Andrei S. Potapov - asp99@yandex.ru

* Corresponding author

Experimental procedures, spectroscopic and analytical data for compounds 1, 3–5.

1-(2-Hydroxyethyl)-3,5-dimethylpyrazole (1). A suspension of 3,5-dimethylpyrazole (4.80 g, 50 mmol) and finely powdered KOH (8.40 g, 150 mmol) in DMSO (15 mL) was vigorously stirred at 80 °C for 1 h. After that, the mixture was allowed to cool to room temperature and 2-chloroethanol (4.03 g, 3.32 mL, 50 mmol) in DMSO (10 mL) was added dropwise during 1 h with stirring and cooling the reaction flask in a water bath. After the addition was complete, heating at 80 °C and stirring was resumed and continued for 20 h. Then the reaction mixture was poured into water (200 mL) and extracted with chloroform (7 × 15 mL). The extract was washed with water, dried over calcium chloride and the solvent was evaporated in vacuo. Product **1** can be purified by crystallization from iPrOH or by vacuum distillation. Yield 88%, colorless

crystals, mp 76–77 °C (lit. mp 75–76 °C [1]), bp 125–127 °C (2.6 kPa). ^{1}H NMR ($CDCl_3$) δ 2.15, 2.19 s (6H, CH_3), 3.89 (t, J = 5.1 Hz, 2H, $PzCH_2CH_2OH$), 4.00 (t, J = 5.1 Hz, 2H, $PzCH_2CH_2OH$), 5.75 s (1H, H4 (Pz)).

1,5-Bis(3,5-dimethylpyrazol-1-yl)-3-thiapentane (3). A solution of 1-(2-tosyloxyethyl)-3,5-dimethylpyrazole (**2**) [2] (6.59 g, 22.4 mmol), $Na_2S \cdot 9H_2O$ (freshly recrystallized from water, 2.68 g, 11.2 mmol) and NaOH (0.18 g, 4.48 mmol) in water (10 mL) was refluxed for 6 h. After the mixture was cooled in a refrigerator a precipitate formed, which was filtered, washed with water and dried. Product **3** was recrystallized from iPrOH. Yield 72%, colorless crystals, mp 82–83 °C (lit. mp 80–81 °C [3]); IR (cm^{-1}): 1550, 1459, 1302 (Pz), 800 (C–S); ^{1}H NMR ($CDCl_3$) δ 2.17, 2.21 (s, 12H, CH_3), 2.79 (t, J = 7 Hz, 4H, $PzCH_2CH_2S$), 4.05 (t, J = 7 Hz, 4H, $PzCH_2CH_2S$), 5.75 (s, 2H, H4 (Pz)); ^{13}C NMR ($CDCl_3$) δ 11.1 (5-CH_3), 13.4 (3-CH_3), 32.1 ($PzCH_2CH_2S$), 48.4 ($PzCH_2CH_2S$), 104.9 (C4 (Pz)), 139.1 (C5 (Pz)), 147.7 (C3 (Pz)); Anal. calcd for $C_{14}H_{22}N_4S$: C, 60.40; H, 7.96; N, 20.12; S, 11.52; found: C, 60.73; H, 8.08; N, 20.44; S, 11.05.

1,8-Bis(3,5-dimethylpyrazol-1-yl)-3,6-dithiaoctane (4). A solution of 1,2-dibromopropane diisothiuronium salt [4] (3.46 g, 10 mmol) and KOH (5.6 g, 100 mmol) in water (15 mL) were refluxed for 5 h. Then, tosylate **2 (**5.88 g, 20 mmol) was added, and refluxing and vigorous stirring were continued for 8 h. After the mixture was cooled to room temperature a precipitate formed, which was filtered and washed with water. Yield 73%, colorless crystals, mp 85–87 °C (iPrOH), lit. mp 78 °C [2]; IR (cm^{-1}): 1549, 1461, 1315 (Pz), 787 (C–S); ^{1}H NMR ($CDCl_3$) δ 2.18, 2.24 (s, 12H, CH_3), 2.52 (s, 4H, SCH_2CH_2S), 2.91 (t, J = 6.6 Hz, 4H, $PzCH_2CH_2S$), 4.10 (t, J = 6.6 Hz, 4H, $PzCH_2CH_2S$), 5.76 (s, 2H, H4 (Pz)); ^{13}C NMR ($CDCl_3$) δ 11.0 (5-CH_3), 13.3 (3-CH_3), 32.0 (SCH_2CH_2S and $PzCH_2CH_2S$), 48.5 ($PzCH_2CH_2S$), 104.9 (C4 (Pz)), 139.1 (C5 (Pz)), 147.7 (C3 (Pz)); Anal. calcd for $C_{16}H_{26}N_4S_2$: C, 56.77; H, 7.74; N, 16.55; S, 18.94; found: C, 56.31; H, 7.65; N, 16.56; S, 18.48.

1,3-Bis(1,2,3-benzotriazol-1-yl)-2-thiapropane (5). A biphasic mixture of 1-chloromethylbenzotriazole [5] (7.82 g, 46.7 mmol), $Na_2S \cdot 9H_2O$ (6.73 g, 28.0 mmol, freshly

recrystallized from water), KOH (2.62 g, 46.7 mmol), and tetrabutylammonium bromide (0.254 g, 2 mol %) in acetonitrile (45 mL) and water (15 mL) was vigorously stirred and refluxed for 12 h. After the reaction mixture was poured into water (150 mL) a precipitate formed, which was filtered, washed with water, dried and recrystallized from acetonitrile. Yield 89%, colorless crystals, mp 182–184 °C (MeCN), lit. mp 179 °C [6]; IR (cm^{-1}): 1612, 1453 (Bta), 750 (C–S); ^{1}H NMR (DMSO-d_6) δ 6.04 (s, 2H, CH_2), 7.44 (t, J = 7 Hz, 2H, H5 (Bta)), 7.56 (t, J = 7 Hz, 2H, H6 (Bta)), 7.94 (d, J = 7.5 Hz, 2H, H4 (Bta)), 8.06 (d, J = 7.5 Hz, 2H, H7 (Bta)); ^{13}C NMR ($CDCl_3$) δ 47.5 (CH_2), 110.9 (C7 (Bta)), 119.2 (C4 (Bta)), 124.3 (C5 (Bta)), 127.5 (C6 (Bta)), 131.8 (C8 (Bta)), 145.2 (C9 (Bta)); Anal. calcd for $C_{14}H_{12}N_6S$: C, 56.74; H, 4.08; N, 28.36.

References

1. 1. Kashima, C.; Tsukamoto, Y.; Higashide, K.; Nakazono, H. *J. Heterocycl. Chem.* **2000,** *37,* 983–990. doi:10.1002/jhet.5570370449
2. Haanstra, W. G.; Driessen, W. L.; Reedijk, J.; Turpeinen, U.; Hämäläinen, R. *J. Chem. Soc., Dalton Trans.* **1989,** 2309–2314. doi:10.1039/DT9890002309
3. García-Antón, J.; Pons, J.; Solans, X.; Font-Bardia, M.; Ros, J. *Eur. J. Inorg. Chem.* **2003,** *2003,* 3952–3957. doi:10.1002/ejic.200300235
4. Speziale, J. A. *Org. Synth.* **1950,** *30*, 35–37.
5. Burckhalter, J. H.; Stephens, V. C.; Hall, L. A. R. *J. Am. Chem. Soc.* **1952**, *74*, 3868–3870. doi: 10.1021/ja01135a044
6. Katritzky, A. R.; Ghiviriga, I.; Oniciu, D. C.; Soti, F. *J. Heterocycl. Chem.* **1996,** *33,* 1927–1934. doi:10.1002/jhet.5570330661
